# Trusting the AI rather than the expert? Evidence from sports-related assessments in relation to the Olympic Games 2024

**DOI:** 10.1007/s00426-026-02409-z

**Published:** 2026-10-30

**Authors:** Fabian Wunderlich, Moritz Reis, Wilfried Kunde, Daniel Memmert

**Affiliations:** 1 Institute of Exercise Training and Sport Informatics, German Sport University Cologne, Cologne, Germany; 2 Institute of Psychology, Julius-Maximilians-Universität Würzburg, Würzburg, Germany

## Abstract

We investigated how predictions regarding the outcome of sports competitions are affected by concurrent predictions which were believed to originate from either a human sports expert or an AI system. Participants were asked to estimate the probability of a certain team to win a group match against the respective opponent in various matches of the 2024 Olympic Games and to report their confidence in that estimate. Moreover, they received numerical estimates for that probability which were either high or low, and which were believed to originate from a human expert or an AI system. Participants’ estimates were biased towards these supplementary estimates, thus reflecting an anchoring effect. Yet, this effect’s size was independent of the believed source of the anchor and even non-significantly higher for AI support. Confidence in provided judgments was even significantly higher when the believed origin of supplementary estimates was AI rather than human. Altogether, the results suggest no bias against AI-generated sports outcome predictions, highlighting the task-dependency of reliance on AI.

**Supplementary Information:**

The online version contains supplementary material available at https://doi.org/10.1007/s00426-026-02409-z .

## Introduction

Artificial Intelligence (AI) has tremendous impact on almost all areas of modern societies, ranging from healthcare to education (Horvitz, 2017). Since the introduction of ChatGPT, AI-based applications, and AI-driven chatbots in particular, have become an even more ubiquitous part of most people’s everyday life. Such tools offer seemingly low threshold access to detailed information on various subjects at low costs. Moreover, they can act as a personalized advisor, providing tailored responses based on the provided input. This includes help with everyday tasks, like giving dining recommendations, but also extents to highly sensitive contexts like providing initial medical assessments based on a provided symptoms report (Haupt & Marks, 2023). Even though AI-generated advice should always be treated with caution due to the risk of hallucination (i.e. false or misleading responses to prompts), the performance of such tools in many domains is remarkable (see e.g., Ayers et al., 2023 for medical advice generation). At the same time, a growing body of research highlights that most people are biased against AI and devalue its advice (Dietvorst et al., 2015). Understanding this so-called *algorithm aversion* is a central goal of psychological research and crucial to overcome unjustified denial of AI use in cases where it would in fact be a very good choice.

Prior research indicates that attitudes toward AI and reliance on AI-generated advice are very task-dependent (Castelo et al., 2019). Thus, it is important to investigate the nature of human-AI interaction in a variety of domains and possible use cases. We here focus on the context of sports in which AI is becoming increasingly important (Naughton et al., 2024). For instance, AI-based digital coaches can generate individualized training schedules and, in combination with corresponding hardware, even provide real-time feedback on workout performance (Jud & Thalmann, 2025). Furthermore, AI systems can analyze vast amounts of data and identify complex patterns beyond human capabilities. Due to these advantages, sports clubs increasingly use AI to predict their athletes’ future performance and prevent injuries (see for a review Claudino et al., 2019). Despite this development, so far, sports have been mainly neglected as a domain to investigate trust in AI (Wunderlich & Reis, 2026).

In the present manuscript, we explore a specific application of AI in sports, namely AI-generated predictions of competition outcomes. Methods to forecast the outcome of a sports competition are of great relevance for various stakeholders (e.g., media, fans, bookmakers, bettors) and thus well-reflected in the scientific literature (see Wunderlich & Memmert, 2021). Yet, the course of any sports match can be affected by countless factors, making it highly challenging to provide a precise prediction for its outcome. Within this context, AI-based tools, which are trained on a huge amount of data, are increasingly used to estimate the probabilities of certain outcomes (e.g., that a given team wins the game; Bunker & Susnjak, 2022; Fialho et al., 2019). Prior research highlights the large potential of AI in this domain, sometimes even outperforming predictions by human experts (e.g., Kahn, 2003). Human users’ perception of AI within this domain, however, has been mainly neglected so far. Here, we thus empirically investigate to which extent human users are influenced by predictions of sports results labelled as AI- compared to human-generated.

Some caution is needed when aiming to objectively assess AI prediction performance of sports results in general, and in relation to humans in particular. Models based on Machine Learning (i.e. AI), for a long time, have struggled to keep up with statistical models in forecasting (Gilliland, 2020). Thus, statistical models are still state-of-the art in sports predictions (Koopman & Lit, 2019), existing in parallel to AI-based models, which increasingly keep up or even outperform statistical models in specific situations (Wunderlich et al., 2025). At the same time human predictions, when aggregated through suitable collaborative mechanisms, have been reported to show a high prediction accuracy (Peeters, 2018; Spann & Skiera, 2009). Predictions of experts, however, have led to poor performance, not being able to outperform laypeople while falling short of a simple prediction rule based on world rankings (Andersson et al., 2005) and were outperformed by sources of collaborative human estimation (Spann & Skiera, 2009). In direct comparisons, AI often performed on an equal level with human experts (Pretorius & Parry, 2016) and sometimes even exceeded human predictions considerably. For instance, in one study an AI tool achieved 75% accuracy in predicting National Football League results compared to only 63% achieved by a team of eight sportscasters (Kahn, 2003). Moreover, even better predictions can be achieved by combining AI with human expertise (Beal et al., 2021).

Despite these objective indications that AI can outperform individual experts in this domain it stands to question whether an AI-generated prediction of a sports result is perceived as equivalent to a prediction by a human expert. Research on *algorithm aversion* (Dietvorst et al., 2015) highlights that most people are skeptical towards AI-generated advice (for a review see Jussupow et al., 2020). Especially when an AI makes a mistake, this mistake is usually perceived as more dramatic than the same error by a human actor (Dietvorst et al., 2015). Algorithm aversion has been documented across various domains, ranging from organizational contexts (Glikson & Woolley, 2020) to healthcare (e.g., Reis et al., 2024). At the same time, previous findings showcase that algorithm aversion is not a universal phenomenon but depends on characteristics of the person, the task and the algorithm (e.g., Castelo et al., 2019; Jussupow et al., 2024). For instance, biases against algorithms are more pronounced when the algorithm is embedded in material (e.g., robots, Kim & Duhachek, 2020) compared to nonmaterial objects (e.g., digital decision aids; Bankuoru Egala et al., 2023), moderated by the stakes which are involved in the respective decision (e.g., medical diagnostics vs. movie recommendations; Filiz et al., 2023) and the extent to which a task is seen as subjective (e.g., dating advice vs. data analysis; Castelo et al., 2019). Importantly, there are even situations in which people may actually prefer algorithmic over human judgement (so-called *algorithm appreciation*; Logg et al., 2019). To reconcile these conflicting findings, recently explanation models have been discussed that might help to understand why in some situations algorithm aversion and in others algorithm appreciation occurs. Kaufmann and Chacon (2026), with reference to Cognitive Continuum Theory, place tasks on a continuum between analytical and intuitive thinking and propose that AI advice is more welcome in analytical tasks. Relatedly, the capability-personalization framework was proposed (Qin et al., 2025). This framework posits that algorithm aversion occurs except when two conditions are simultaneously fulfilled: (a) the algorithm is perceived as more capable than humans in this task and (b) personalization is perceived as unnecessary in the given context. Only under these two conditions algorithm appreciation should arise. Specific for sports-related contexts research on the public’s perspective on AI is surprisingly scarce (Wunderlich & Reis, 2026). The limited, questionnaire-based evidence highlights that most people have overall positive attitudes towards the use of AI-based fitness tools (Chin et al., 2022). Positioning sports within the two dimensions of the capability-personalization framework by Qin and colleagues (2025) is challenging. On one side, estimating the outcome of sports matches is a strongly data-driven use case, with little need for personalization. On the other side, sports are also a domain often seen as highly emotional, where AI may be judged as less capable than a human expert. Accordingly, it is difficult to derive clear predictions on whether algorithm aversion or appreciation should manifest in this context and additional research on this question is urgently needed.

In the present research we relied on a rather implicit and behavioral measure of trust in AI. The basic phenomenon we employed is called *anchoring effect*. Numerical estimates for an unknown value are typically biased towards additional numerical references that are made available to the estimating person (Tversky & Kahneman, 1974). This is one of the most robust findings in human decision-making (Furnham & Boo, 2011). It is prevalent across a variety of domains like general knowledge (McElroy & Dowd, 2007) and probability estimates (e.g., estimating the likelihood of a nuclear war; Plous, 1989); and can also be observed in several real-world settings (e.g., legal judgements; Mussweiler & Englich, 2005). Moreover, anchor-induced judgment biases increase with perceived relevance of the anchor (Glöckner & Englich, 2015; van Exel et al., 2006), specifically when the judgement is of low criticality (Johnson & Johnson, 2017). For instance, hypothetical sentencing decisions were more affected by relevant anchors (i.e., the opinion of a professional judge on the present case) than irrelevant ones (i.e., the opinion of a lay judge on a completely unrelated case; Glöckner & Englich, 2015). We thus suggest that any difference of anchoring effects depending on whether the source of this anchor is believed to be human or an AI indicates perceived relevance of this source for the required judgement. While anchoring effects are also subject to other influences (see for an overview, Furnham & Boo, 2011), situational or interindividual factors like mood, personality or expertise would have highly limited influence on the anchoring effect in the present experimental design given that the same participants are provided with both believed sources of the anchor. Still, not all additional factors possibly influencing the anchoring effect can be controlled for, thus our interpretation of the anchoring effect as a proxy for algorithm aversion has an exploratory character and should be validated in future research.

To explore the present research question, we conducted an online experiment, in which participants were asked to predict outcomes of competitions in the 2024 Olympic Games. We aimed to reduce possible discipline-specific effects by investigating predictions in three different sports, namely football, handball and field hockey. To test the effect of algorithmic and human advice, we provided participants with additional predictions labelled as either coming from an AI or a human expert. Provided predictions were either somewhat higher (Exp+, AI+) or lower (Exp- and AI-) than a realistic estimation. We tested the following hypotheses:

### Hypothesis 1

Given the robust evidence for the existence of anchoring effects described above, we assume that they are likewise present in our specific experimental task and hypothesize that participants are influenced by the additional information, i.e. provide higher estimations if presented with a higher reference value (conditions Exp + and AI+) than with a lower reference value (conditions Exp- and AI-).

### Hypothesis 2

Moreover, we assume that the strength of this anchoring effect differs when the additional information is labelled as stemming from a human expert compared to an AI. Given the inconclusive findings on aversion and appreciation of algorithmic advice (e.g., Dietvorst et al., 2015; Logg et al., 2019), we do not specify a clear direction for this hypothesis and treat it as rather exploratory. Consequently, we hypothesize that the additional information affects participants to varying degrees depending on if it is labelled as stemming from an expert (conditions Exp + vs. Exp-) compared to an AI (conditions AI + vs. AI-).

### Hypothesis 3

Moreover, trust in an advice source is positively linked to confidence in the final decision (Sniezek & van Swol, 2001). Following from Hypothesis 2, we thus hypothesize that participants report different levels of confidence in their prediction when the additional information is labelled as stemming from a human expert (conditions Exp + and Exp-) compared to an AI (conditions AI + and AI-).

## Methods

### Experimental procedure and design

We conducted an online experiment with three versions, each asking participants to conduct an online study with regard to one of three men’s team sports competitions (football, handball, field hockey) of the Olympic Games 2024 in Paris. These sports were chosen as they all have a group stage where teams play against each other in a predefined schedule (allowing forecasts for all these matches to be made before the start of the Olympic Games) and they are popular sports in Germany, where the study was conducted. Participants were asked to provide personal information on their age (in years), gender (male/female/other) and self-assessed expertise in the respective sport (scale of 1 to 10 with a higher value meaning higher expertise). Then, they were asked to estimate the probability (in percent) of a team to win a group match (against the respective opponent) and to state how sure they were in their assessment (on a scale ranging from 1 = *very unsure* to 6 = *very sure*) for a multitude of matches. For each match, the participants either received no additional information or a probability labeled as an assessment of an expert or a probability labeled as an assessment of an AI. The additional information did not really stem from an expert or an AI, but was deliberately controlled to either display a slightly higher or slightly lower value than a realistic estimate (see Section "Determination of the probability information labelled as AI or Expert" for more details). This sums up, to a total of five different conditions (denoted as No, Exp+, Exp-, AI+, AI-), of which one was randomly assigned to each participant for each of the matches. Naturally, the participants knew which additional information they were provided with, but were unaware of whether they received a deliberately higher or lower value. As the very last question, participants were asked to state which additional information (expert, AI or none of both) they considered the most helpful for their estimations.

The study was implemented in German language and opened only a few days before the start of the Olympic Games to ensure a direct temporal connection to the tournament. Participants had the possibility to complete the study between 17th of July 2024 and 23rd of July 2024 for football (tournament start: 24th of July 2024), between 16th of July 2024 and 26th of July 2024 for handball (tournament start: 27th of July 2024) and between 18th of July 2024 and 26th of July 2024 for field hockey (tournament start: 27th of July 2024) via SoSci Survey (Leiner, 2024).

For each sport, the study included 24 matches, representing all group matches in football and the first 24 of 30 group matches in handball and field hockey. Data for two matches were excluded from the final analysis due to manual errors in the study design, where for match 3 in football (Egypt vs. Dominican Republic) an input field was displayed twice in one condition and for match 10 (South Africa vs. Great Britain) in field hockey the wrong of the two teams was named in the question, leading to an erroneous probability estimation by expert / AI. Consequently, the final results are based on 70 matches. The present study has been officially approved by the Ethics Committee of the German Sport University Cologne. All participants have stated their consent to participate.

### Determination of the probability information labelled as AI or expert

**Fig. 1:**
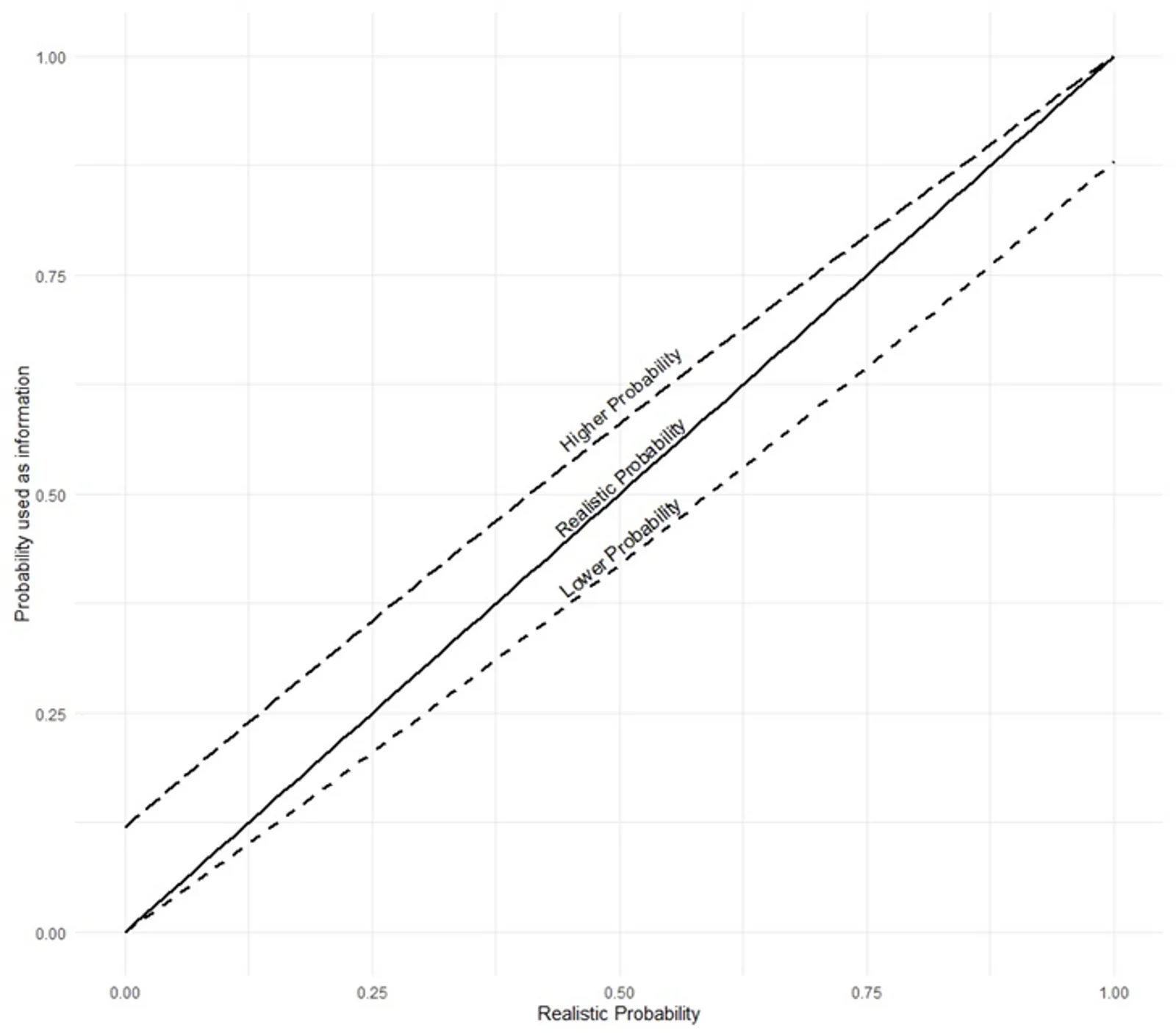
Illustration of lower and higher probability displayed to the participants in the lower (AI-/Exp-) and higher (AI+/Exp+) conditions as a function of the realistic outcome probability

The design required us to provide information on probabilities being deliberately higher or lower than a realistic value, which is tricky, as no ground truth on the probability of a match outcome is known prior to the matches. Betting odds (i.e. market prices from the sports betting market) can easily be transferred to outcome probabilities and have been demonstrated to be highly accurate estimations (Forrest et al., 2005; Wunderlich, 2024; Wunderlich & Memmert, 2018), however betting odds were not available for all matches before the start of the tournaments, yet. Thus, we used the following procedure: We obtained betting odds for the tournament winner (which were already available) as an objective assessment of team strengths and derived a realistic winning probability consistent with these strengths for each match (i.e. question). Then, we transformed these realistic probabilities into slightly higher and slightly lower probabilities displayed as information stemming from the AI or expert in our experimental conditions. Fig. 1 illustrates the way higher and lower probabilities were set in relation to the realistic one, where no probabilities are set lower than 0% or higher than 100% and the average probability difference between the higher and lower condition is roughly 15%. To ensure that our estimations had a sufficiently high accuracy to be used in the experiments, we tested this after the tournaments, when information on the actual match outcomes and the betting odds for each match were available, confirming that there was no bias in the order of magnitude of the probability estimations (average own estimation: 44.65%; average estimation from betting odds: 44.83%) and the error (measured as RMSE) of the own estimation to the actual match outcomes was comparable to the one from the betting odds, but better than the estimations of the participants in the No-Condition. For more detailed information on the determination of probability information and results of accuracy tests, we refer to the Supplementary Material, Part A.

### Inclusion and exclusion criteria

The online study was open to any person willing to participate in it and considered adult in the country of execution (at least 18 years). However, useful assessment of the teams probably requires at least some basic knowledge in the respective sport. Thus, the study link was particularly shared among persons being actively involved in one of the respective sports by contacting coaches and team members and sharing the links via mailing lists with reference to the respective sport. In total, there were 384 persons (football: 102, handball: 161, field hockey: 121) accessing the study. We excluded datasets of 44 persons (football: 13, handball: 21, field hockey: 10) not indicating an age of at least 18 or not formally approving their consent to participate. Moreover, we excluded datasets of 64 persons (football: 18, handball: 39, field hockey: 7) not giving assessments for at least half of the matches included in the study. Finally, we excluded datasets of 3 persons (football: 1, handball: 2) conducting the study after the tournament had already started. As a result, *N* = 273 (football: 70, handball: 99, field hockey: 104) participants were involved in the study.

### Statistical analysis

#### Descriptive statistics and x^2^-tests

For all non-match specific questions (i.e. personal characteristics and general questions), we report descriptive statistics separated by experiment reporting mean, standard deviation (SD), minimum (min), maximum (max) as well as median for ordinal variables to provide an overview on the participants and their differences across experiments. Moreover, we analyse whether participants’ answers to non-match specific questions (self-assessed expertise and which information was most helpful) depend on their personal characteristics (age and gender). We do so by performing and reporting $\:{\chi\:}^{2}$-tests testing for a relationship between all combinations of questions and personal characteristics. In these (and all following) analyses, we consider p-values of lower than 5% to be statistically significant.

#### Hypothesis testing (Linear Mixed Model)

**Fig. 2:**
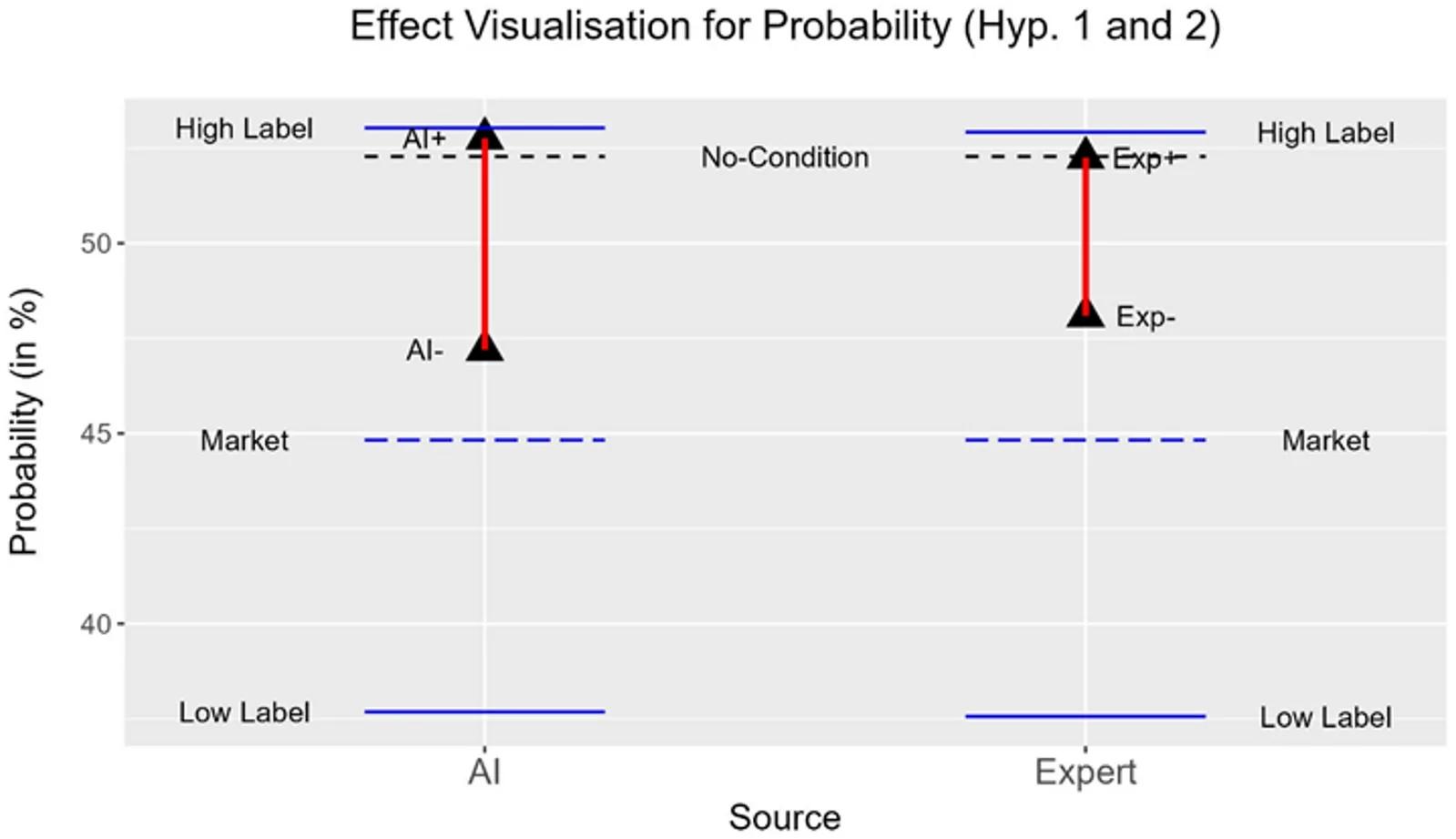
Illustration of the relevant effects with regard to Hyp. 1 and 2. Blue lines refer to the average probability displayed in the Exp + and AI+ condition (denoted as High) and in the Exp- and AI- condition (denoted as Low) as well as the market estimation obtained from betting odds as a realistic probability estimation. The dashed black lines refer to the (slightly biased) average estimation in the No-condition. Triangles illustrate the average estimations in each of the four remaining conditions, while the red lines indicate the anchoring effects

**Fig. 3:**
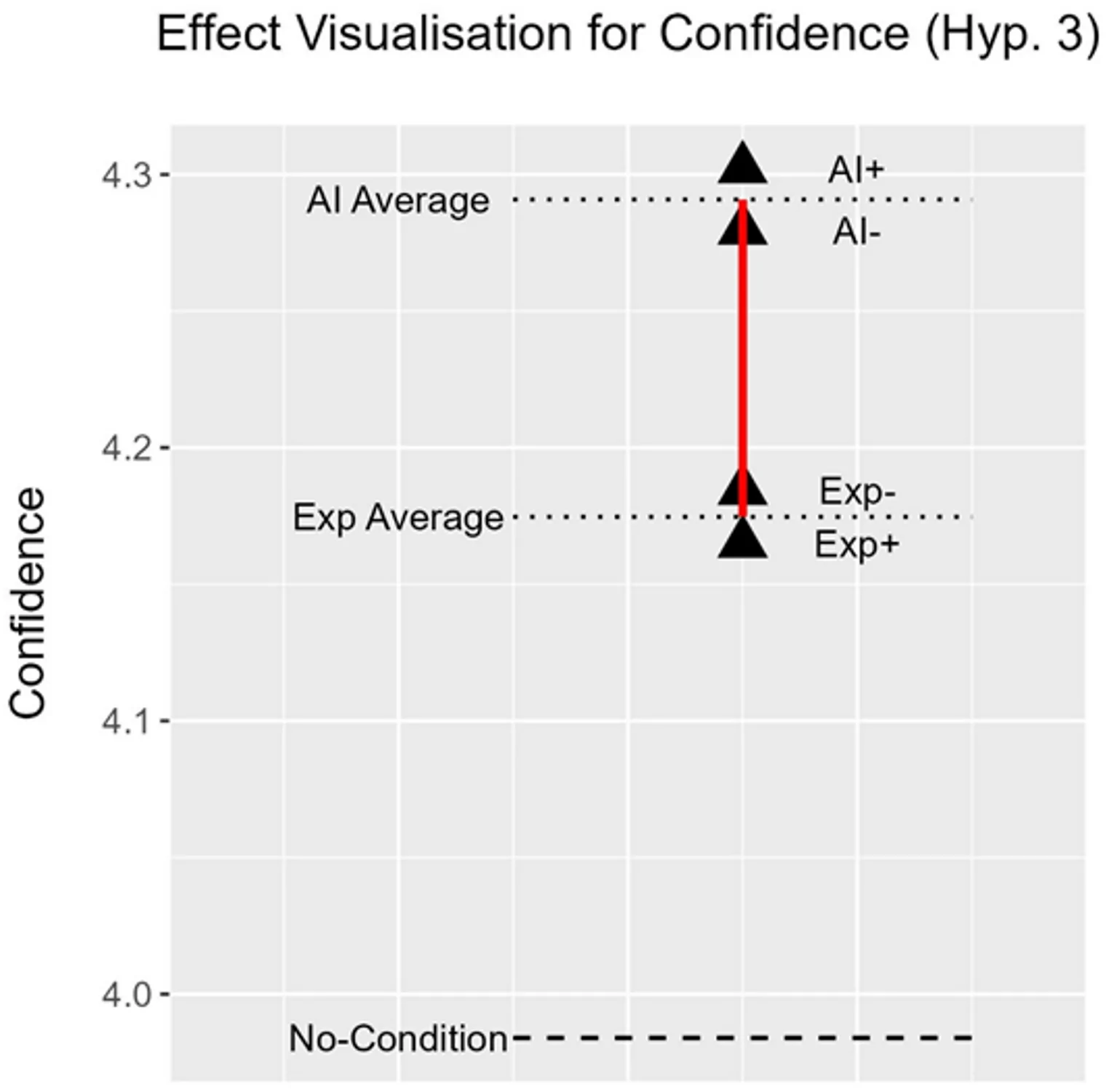
Illustration of the relevant effect with regard to Hyp. 3. The dashed black lines refer to the average confidence in the No-condition. Triangles illustrate the average confidence in each of the four remaining conditions, dotted lines the average confidence in both AI conditions or Exp conditions respectively and the red line indicates the effect of the source label on confidence

The structure of the data is highly nested as repeated measurements of the same matches (across different participants) and the same participants (across different matches) occur. To account for this, linear mixed-effects models are used to test the study’s hypotheses. Our design involves five conditions, where we use the No-condition only as a baseline (see Section "Determination of the probability information labelled as AI or Expert" or Figs. 2 and 3). The remaining four conditions can be modelled as two independent variables, namely the Source (AI or Expert) and the Level (lower or higher value) of the information, whose interaction is also included in the model. We code the values for Source as AI = -0.5 and Exp = + 0.5 and for Level as lower (“-“) = -0.5 and higher (“+”) = + 0.5. Thus, the model estimate for Level can be directly interpreted as the anchoring effect (in %) and the interaction estimate can be directly interpreted as the difference between the anchoring effects for Expert and AI (in %). For Hypothesis 1 and 2, the winning probability estimated by the participants (denoted as Probability) is the dependent variable and the full model, reads as follows:

*Full Model*^1^:

$$\begin{aligned}{Probability}_{jk}&={\beta\:}_{0}+{\beta\:}_{1}{Level}_{jk}+{\beta\:}_{2}{Source}_{jk}\\&+{\beta\:}_{3}\left({Level}_{jk}\:x\:{Source}_{jk}\right)\\&+{u}_{j}+{v}_{k}+{\epsilon\:}_{jk}\end{aligned}$$

The index j represents the participant and k the match in each trial, where each combination of j and k can only occur once, i.e. no participant gives an estimation twice for the same match. The random intercepts $\:{u}_{j}$ and $\:{v}_{k}$ allow individual intercepts for each participant and match. The remaining part equals a linear regression model including Level, Source and its interaction with $\:{\epsilon\:}_{jk}$ being the error term. Hypothesis 1 relates to a significant (positive) effect of Level on Probability, while Hypothesis 2 relates to a significant interaction between Source and Level. A significant effect of Source would mean that the AI or Expert label itself systematically affects the estimation, which neither matches the focus of this study nor would be expected. We test significance of the models by performing likelihood ratio tests comparing the fit of a model including the respective effect to a model lacking the respective effect, defining:

*Restricted Model (no interaction effect)*:

$$\begin{aligned}{Percentage}_{jk}&={\beta\:}_{0}+{\beta\:}_{1}{Level}_{jk}\\&+{\beta\:}_{2}{Source}_{jk}+{u}_{j}+{v}_{k}+{\epsilon\:}_{jk}\end{aligned}$$

*Null Model (no effect of Level)*:

$$\:{Percentage}_{jk}={\beta\:}_{0}+{\beta\:}_{1}{Source}_{jk}+{u}_{j}+{v}_{k}+{\epsilon\:}_{jk}$$

To answer Hypothesis 3, we use similar, but slightly modified models, where the self-assessed confidence of the participants (denoted as Confidence) is the dependent variable. Please note that this procedure involves a simplification as we assume the differences between the six levels of confidence (in the actually ordinal variable) to be equidistant. In this case, the models read as follows:

*Full Model*:

$$\begin{aligned}{Confidence}_{jk}&={\beta\:}_{0}+{\beta\:}_{1}{Level}_{jk}\\&+{\beta\:}_{2}{Source}_{jk}+{u}_{j}+{v}_{k}+{\epsilon\:}_{jk}\end{aligned}$$

*Null Model (no effect of Source)*:

$$\:{Confidence}_{jk}={\beta\:}_{0}+{\beta\:}_{1}{Level}_{jk}+{u}_{j}+{v}_{k}+{\epsilon\:}_{jk}$$

Hypothesis 3 relates to a significant effect of Source on Confidence, while we do not expect an effect of Level and do not include an interaction effect to the models.

#### Power analysis

We performed a simulation-based power analysis estimating the probability to correctly reject the null hypothesis for a variety of realistic scenarios of effects and variances in the data. We found that the model would be able to detect effects being as small as one tenth of the residual model standard deviation with sufficient power for Hypothesis 1 and 3. Power is more limited for the interaction effect related to Hypothesis 2, but here, at least effects as small as one fifth of the residual model standard deviation would be detected with a power exceeding 80%. Based on these analyses, we considered the design to have sufficient power to detect relevant effects. For all details on the specific considerations of power analysis in linear mixed models in general and our specific design in particular as well as all assumptions taken and results obtained, we refer to the Supplementary Material, Part B.

#### Exploratory analyses

The models to investigate the three main hypotheses do not yet involve further variables from our dataset such as personal characteristics of the participants. Although it would be interesting to know, e.g. whether the gender of participants significantly influences the effects of interest, consideration of additional variables in the models would require inclusion of several interaction terms, decreasing model interpretability, potentially affecting model convergence and like in any investigation of interactions imply decreased statistical power. For this reason, we do not include further variables to the models, but we repeat model analyses on specific subsets of the data distinguished by the three experiments (i.e. sports), gender, age group, self-assessed expertise and own assessment on which additional information was most helpful. Despite reporting results of inferential statistics, these analyses have a highly exploratory nature and need to be treated with caution for several reasons: First, we do not test whether the additional variables significantly affect the effects of interest, but only whether the effects of interest are significant in each subgroup. Second, we did not and in some cases are not able to obtain theory-backed hypotheses for which variables should theoretically take an influence. Third, the personal characteristics and answers to questions might be highly dependent on each other leading to selection effects. Fourth, multiple testing of various subgroups increases the risk of false significant results.

### Transparancy and openness

All data and code of analyses were made publicly available online. A simulation-based analysis of power, all exclusions of data (i.e. participants), all manipulations and all measures in the study have been reported transparently in the Methods section. All statistical analyses and visualisation were performed in R Statistical Software version 4.3.2 (R Core Team, 2023) using the packages lme4 (Bates et al., 2015), simr (Green & MacLeod, 2016) and ggplot (Wickham, 2016), among others. This study’s design and its analyses were not preregistered.

## Results

### Descriptive statistics

Participants taking part in the study were 32.81 ± 13.22 years old (minimum: 18, maximum: 67). 71.7% of participants identified as male and 27.6% as female (remaining 0.7%: no answer). The median expertise was 7 on a scale from 1 indicating lowest expertise to 10 indicating highest expertise. Interestingly, after having answered the match-specific questions, the majority of participants (51.1%) stated that none of the additional information had been helpful, while only 17.3% found the AI and 29.0% found the expert to be most helpful (2.6%: no answer). The structure of age, gender and expertise was relatively homogeneous across all three sports. Please find all descriptive statistics for non-match-related questions and further separated by experiment in Table 1.

**Table 1:** Descriptive statistics for the non-match-related variables

	Age				Gender			Expertise			Helpful			
	Mean	SD	Min	Max	Male	Female	No answer	Median	Min	Max	Expert	AI	None	No answer
Football	40.09	16.39	19	67	45 (64.3%)	24 (34.3%)	1 (1.4%)	6	1	10	17 (24.3%)	9 (12.9%)	41 (58.6%)	3 (4.3%)
Handball	31.37	12.08	18	62	64 (65.3%)	33 (33.7%)	1 (1.0%)	7	1	10	26 (26.5%)	12 (12.2%)	57 (58.2%)	3 (3.1%)
Field Hockey	29.28	9.58	18	63	86 (82.7%)	18 (17.3%)	0 (0.0%)	7	1	10	36 (34.6%)	26 (25.0%)	41 (39.4%)	1 (1.0%)
All	32.81	13.22	18	67	195 (71.7%)	75 (27.6%)	2 (0.7%)	7	1	10	79 (29.0%)	47 (17.3%)	139 (51.1%)	7 (2.6%)

### Interindividual differences

Table 2 reports results of testing whether the personal characteristics (gender and age) of participants showed a relation to their answers regarding self-assessed expertise and information perceived as most helpful. Results show a significant relationship between gender and self-assessed expertise (*p* < .001), where male participants tended to state a higher expertise and between gender and the more helpful information (*p* < .05), where male participants more often indicated that AI was the most helpful information compared to female participants. Moreover, we find a significant relationship between age group and the most helpful information (*p* < .01), where younger participants tended to be more likely to indicate that AI was helpful. We do not find a significant relationship between age group and self-assessed expertise (*p* = .09), but a tendency for younger participants to more often indicate a higher expertise. For more detailed information on the frequencies, please refer to the Supplementary Material, Part C.

**Table 2:** Results of chi-squared tests investigating relationships between personal characteristics of participants and their answers in the study

Personal Characteristics	Participant answers	$\:{\chi\:}^{2}$	df	*p*-value
Gender	Expertise	32.90	9	< 0.001*
Gender	Helpful Information	8.79	2	< 0.05*
Age	Expertise	37.41	27	.09
Age	Helpful Information	22.23	6	< 0.01*

*Statistically significant relationship

### Anchoring effect and human- vs. AI-labelled information (Hypothesis 1 und 2)

Figure 2 shows the relevant effects with regard to Hypotheses 1 and 2 summarised across all matches in all three experiments (sports). The anchoring effect (emphasised by red lines) being consistent with Hypothesis 1 is clearly visible. Given that this effect is slightly higher for the AI conditions, we can infer that, in relation to Hypothesis 2, participants tended to be slightly more influenced by the information labelled as AI. Given the baseline of market prices, we can see that the deliberately higher and deliberately lower information given in the study indeed was realistic. However, the Figure reveals a bias of participants tending towards a too high probability estimation (baseline of the No-condition), presumably ignoring that the probability of teams winning is slightly lower than 50% driven by the possibility of draws.

Model estimates of the linear mixed model are displayed in the upper part of Table 3. They indicate an effect of Level (i.e. an anchoring effect as hypothesised in Hypothesis 1) of 4.75%. With regard to Hypothesis 2, which relates to the interaction coefficient, the model shows a tendency of participants to be more influenced by the AI than by the expert, where the anchoring effect for Experts is 1.32% lower than for AI. The results of likelihood ratio tests formally testing our hypotheses for significance can be found in the lower part of Table 3. The restricted model including Level provides a better fit for the data than the null model without it ($\:{\chi\:}^{2}\left(1\right)$ = 103.12, *p* < .001), statistically confirming Hypothesis 1. The full model additionally including an interaction of Level and Source did not provide a statistically significant improvement to the restricted model ($\:{\chi\:}^{2}\left(1\right)$ = 2.01, *p* = .156). Thus, Hypothesis 2 is not supported, while the effect estimate shows a tendency towards the AI. The random variances indicate that participants and matches explain a considerable proportion of the variance in the probability estimations, where the random variance of Participant is negligible while the random variance of Match even exceeds the residual variance. The latter can be interpreted as a direct consequence of the natural dispersion of team strengths reflected in the participants’ estimations.

**Table 3:** Coefficients and variances estimated in the linear mixed model for Probability (Hyp. 1 and 2). The upper part of the Table refers to the model estimates and random effects while the lower part refers to the results of likelihood ratio tests formally testing hypotheses by comparing the fit of model versions including the relevant variable to restricted model versions excluding the relevant variable

Model Estimates – Full Model (Probability)			
*Fixed Effects*			
	Estimate	Std. Error	
Intercept	49.92	2.72	
Level	4.75	0.46	
Source	0.33	0.47	
Level: Source	-1.32	0.93	
*Random Effects*			
Groups	Name	Variance	Std. Dev.
Participant	Intercept	11.51	3.39
Match	Intercept	511.28	22.61
Residual		260.50	16.14
Results of Likelihood Ratio Tests			
***Hypothesis***	***Effect***	***Model Comparison***	***Test result***
Hyp. 1	Level	Restricted vs. Null	$\:{\chi\:}^{2}\left(1\right)=103.12,\:p<.001*$
Hyp. 2	Interaction	Full vs. Restricted	$\:{\chi\:}^{2}\left(1\right)=2.01,\:p=.156$

*Statistically significant effects

### Influence of human or AI advice on confidence

Figure 3 illustrates the relevant effect with regard to the self-assessed confidence. The red line indicates that, in consistency to Hypothesis 3, the self-assessed confidence was dependent on the source label, where we find it to be higher when provided with information labelled as AI than if provided with information labelled as expert. The baseline of the No-Condition reveals that (independent of the source), participants stated a higher confidence if provided with an additional information.

Model estimates of the linear mixed model are displayed in the upper part of Table 4, where the model estimates an effect of Source increasing the self-assessed confidence by about 0.1 when provided with information labelled as AI. The full model on Confidence including Source provides a better fit for the data than the null model without it ($\:{\chi\:}^{2}\left(1\right)$ = 18.65, *p* < .001), statistically confirming Hypothesis 3. Again, the random variances indicate, that participants and matches explain a considerable proportion of the variance in the dependent variable, where Participant explains more variance than Match, suggesting that the self-assessed confidence has a stronger dependency on the participant (and its personal characteristics) than on the situation (i.e. the specific match).

**Table 4:** Coefficients and variances estimated in the linear mixed model for Confidence (Hyp. 3). The upper part of the Table refers to the model estimates and random effects while the lower part refers to the results of a likelihood ratio test formally testing the hypothesis by comparing the fit of full model and null model

Model Estimates – Full Model (Confidence)				
*Fixed Effects*				
	Estimate		Std. Error	
Intercept	4.20		0.07	
Level	0.02		0.02	
Source	-0.10		0.02	
*Random Effects*				
Groups	Name		Variance	Std. Dev.
Participant	Intercept		0.58	0.76
Match	Intercept		0.19	0.44
Residual			0.61	0.78
Results of Likelihood Ratio Tests				
***Hypothesis***	***Effect***	***Model Comparison***		***Test result***
Hyp. 3	Source	Full vs. Null		$\:{\chi\:}^{2}\left(1\right)=18.65,\:p<.001*$

*Statistically significant effects

### Exploratory analyses

Table 5 summarises results of the exploratory analyses by reporting the estimated model effect and the related p-value relevant to all three hypotheses (Hyp. 1: the effect of Level on Probability, Hyp. 2: the interaction effect of Level and Source on Probability, Hyp. 3: the effect of Source on Confidence) when fitting the full models to several subgroups. The anchoring effect is highly robust against choosing specific subgroups and shows significance for each subgroup. The model coefficients for the anchoring effect are highest for participants in the younger age group, indicating a lower expertise as well as for those indicating AI or expert as the most helpful information. Moreover, we find the anchoring affect to be larger in field hockey than in the other sports. The only subgroup with a significant interaction effect, i.e. being significantly more influenced by the AI information than by the expert information, are the participants who indicated AI as the most helpful information. In all subgroups except for handball, however, we at least find a non-significant tendency towards the AI information. Estimates suggest the smallest interaction effects for participants being female, indicating lower expertise or indicating the expert as the most helpful information. This also applies to participants in the handball experiment when compared to the other two experiments. With regard to the self-assessed confidence, we find significant differences towards AI in field hockey, for male participants, participants with higher expertise, in the younger age group and for participants indicating AI as the most helpful information. Even participants reporting the expert as the most helpful information, indicate a higher confidence when provided with AI information (only closely failing to reach significance). The only subgroups showing no tendency towards AI are female participants and participants in the handball experiment. As we did not formally test significance of differences between subgroups, nor specifically designed the experiments to allow for such an analysis, the results remain exploratory and require cautious interpretation. More detailed results, i.e. all model estimates and full results of likelihood ratio tests can be found in the Supplementary Material, Part D.

**Table 5:** Results of exploratory analyses reporting the estimated effects and related *p* values for all three hypotheses when repeating the above analyses for specific subgroups of the participants. Please note that N refers neither to the number of persons, nor to the number of matches, but to the number of individual estimations of a match

Subgroup		*N*	Hyp. 1: Effect of Level on Probability		Hyp. 2: Interaction Effect on Probability		Hyp. 3: Effect of Source on Confidence	
			Model Estimate	*p*-value	Model Estimate	*p*-value	Model Estimate	*p*-value
All		4948	4.75	*p* < .001*	-1.32	*p* = .156	-0.10	*p* < .001*
Sport	Football	1253	4.23	*p* < .001*	-3.12	*p* = .128	-0.02	*p* = .663
	Handball	1814	2.95	*p* < .001*	0.43	*p* = .781	0.04	*p* = .286
	Field Hockey	1881	6.69	*p* < .001*	-1.70	*p* = .210	-0.29	*p* < .001*
Gender	Female	1322	4.50	*p* < .001*	-0.20	*p* = .921	0.01	*p* = .814
	Male	3590	4.79	*p* < .001*	-1.49	*p* = .158	-0.14	*p* < .001*
Expertise	Higher ( > = 7)	2755	4.13	*p* < .001*	-1.68	*p* = .170	-0.11	*p* < .001*
	Lower ( < = 6)	2193	5.86	*p* < .001*	-0.47	*p* = .740	-0.08	*p* < .05*
Helpful Information	AI	858	8.64	*p* < .001*	-5.36	*p* < .01*	-0.30	*p* < .001*
	Expert	1426	5.88	*p* < .001*	-0.23	*p* = .901	-0.08	*p* = .073
	None	2589	2.70	*p* < .001*	-0.64	*p* = .627	-0.04	*p* = .193
Age Group	< 30	2970	5.29	*p* < .001*	-0.80	*p* = .482	-0.15	*p* < .001*
	>= 30	1978	3.62	*p* < .001*	-1.77	*p* = .265	-0.02	*p* = .652

*Statistically significant effects

## Discussion

The present study examines whether predictions of sports match outcomes are more strongly influenced by information believed to come from a human expert or an AI. To do so, we asked participants to estimate the winning probabilities for matches of the 2024 Olympic Games in Paris (handball, football and field hockey). Before making their judgment, participants received numerical estimates for these probabilities, labelled as stemming from a human expert or an AI. We observed that our participants’ predictions were biased towards these estimates, thus reflecting an anchoring effect. Yet, this bias was of similar size irrespective of whether the believed source was a human expert or an AI, even reflecting a non-significant tendency in favour of the AI. Moreover, participants reported higher confidence in their own predictions when supplemented by a prediction from an AI rather than a human expert. Altogether, the present results provide no evidence for aversion against AI-generated predictions of sport event outcomes.

The bias in participants’ probability judgements driven by the provided estimates is in line with Hypothesis 1 and consistent with a large amount of previous evidence on anchoring effects (e.g., Furnham & Boo, 2011; Mussweiler & Englich, 2005; Tversky & Kahneman, 1974). We observed this effect across all sports disciplines, expertise levels and age groups. Consistent with previous findings, the size of this bias was reduced for participants who stated that the additional estimates were not helpful for their judgements (see Glöckner & Englich, 2015). Yet, even for these participants, a significant anchoring effect was present, thus highlighting a gap between perceived relevance and actual reliance on the anchor (see also Meub & Proeger, 2015). Overall, it is remarkable that the majority of participants judged neither of the additional estimates as helpful for the task, even though the provided values were within a realistic range. This outcome, however, may also reflect some kind of hindsight bias (see for a meta-analysis Guilbault et al., 2004). Participants might assume, consciously or unconsciously, that they would have provided the same estimate, even without the additional information.

Within the present study, we used the size of this anchoring effect, as an implicit measure for trust in the external advice. This assumption is rooted in previous evidence which indicates an influence of believed trustworthiness of the source of an information on respective anchoring effects (e.g., Glöckner & Englich, 2015). To capture potential AI aversion, we thus analysed whether the size of the anchoring effect, i.e., the extent participants relied on the external information, was moderated by the different source labels (human expert vs. AI system). However, in contrast to Hypothesis 2, anchoring effects did not differ significantly between the human compared to the AI condition. This result is consistent with Gültekin and Siniksaran (2025) reporting a similar result (no significant difference, but a tendency towards compliance with AI rather than human advice) in a different, but related task on predicting football matches. As participants’ decisions had no personal consequences this may have reduced a potential influence of the source label. Overall, our results however suggest that algorithm aversion is no universal phenomenon but highly task-dependent (see also Castelo et al., 2019; Jussupow et al., 2024). In the present case, aspects which may have favoured the emergence of algorithm aversion (e.g., high emotionality of the sports context) and factors which could have led to algorithm appreciation (e.g., low need for personalization) may have balanced each other out. In addition, due to the rapid expansion of AI-based tools also human attitudes towards AI may change over time (see e.g., Zehnle et al., 2025). The inherent uncertainty that often drives biases against novel technologies may thus already be reduced (see Mueller et al., 2012; Reis & Kunde, 2024) and perceived benefits of these advancements may have become increasingly salient (see also Reis et al., 2025). Note, however, that this explanation is speculative as we did not directly measure these mechanisms within the present experiment. Remarkably, when explicitly asked which advice source participants experienced as more helpful, there was a clear preference for the human expert, especially for older participants (≥ 30 years). These divergent findings highlight a discrepancy between stated and revealed preferences, which aligns with previous studies in the context of human-AI interaction (e.g., Böhm et al., 2023; Leib et al., 2024).

In line with Hypothesis 3, participants’ confidence ratings differed significantly between both conditions. That is, they were significantly more confident in their judgements in the AI compared to the human expert condition. This outcome may be related to the general tendency of participants to rely more on the AI advice (see Hypothesis 2). That is, confidence in a final decision has been found to be positively influenced by trust in the advice source (Sniezek & van Swol, 2001). If participants in general had more trust in the AI (at least descriptively), this may explain why they were also more confident in their final judgements compared to the human expert condition. At the same time, the label manipulation may have different psychological effects among participants. For instance, in the context of a rather analytical task, the AI label could also evoke greater perceptions of objectivity, precision or statistical accuracy (which in turn may lead to more trust in the AI-labelled information).

Exploratory analyses of subgroups of participants yielded further interesting observations, with gender differences being particularly noticeable. Female participants very rarely considered AI the most helpful information, did not show increased confidence in their estimations when provided with an AI information (in contrast to male participants) and showed no considerable tendency to be more influenced by AI than by experts. In general, females stated a significantly lower expertise than males, however, in light of previous research (González-Betancor et al., 2019; Pallier, 2003) this might fully or partially be caused by differences in self-assessment rather than objective expertise. In this context, it needs to be acknowledged that both self-assessed expertise and perception of AI could have been influenced by the fact that the experiment focused on men’s competitions for all three sports (handball, football, field hockey). Thus, the question of how gender influences the assessment of AI and whether such differences particularly apply in sports-specific tasks will require specific experimental designs in future work.

In general, studies designed like the present one can suffer from trade-offs as both explicit and implicit (i.e. behavioral) measures of trust in the source of an information are subject to disadvantages. Explicit measures, i.e. self-assessed trust is susceptible to social desirability biases, and it remains unclear whether self-assessed trust and the degree of influence on one’s own behaviour align. The present study thus deliberately focused on a rather implicit measure by investigating the actual influence of a source of information on the estimation behaviour of participants. The size of the anchoring effect as an implicit measure of trust, while backed up by previous research (Glöckner & Englich, 2015), remains somewhat ambiguous as it cannot be ruled out that the size of an anchoring effect might potentially also be influenced by further factors besides trust. However, in any case, this measure provides a close estimate of the influence of the source label effect (human vs. AI) on participants’ judgements.

Despite thoughtfully designing the experiment, some weaknesses and limitations have to be acknowledged. Given the within-subject design, participants were confronted with all sources of information (AI, expert or none) and might have been able to suspect the present research question. However, such a demand effect would likely be reflected in a bias against the algorithmic advice, which was not the case in our study. Furthermore, while our used source label manipulation is commonly applied in research on human-AI interaction (see for an overview, Qin et al., 2025), it may constitute a relatively weak manipulation of advice source. Not all participants may have believed that this manipulation is real and thus may have treated both source labels as largely equivalent, which could explain the non-significant influence of the advice source on the anchor effect. Moreover, in light of the results of the No-condition, it may be the case that anchors were chosen in a suboptimal way, as the higher probability assessments in the Exp + and AI+ conditions were already in line with participants’ estimations if not provided with any additional information. As such, the anchoring effect arguably is mainly attributable to the Exp- and AI- conditions reducing participants' original estimations (see also Fig. 2). Thus, if compared to the case of anchors set in perfect relation to the estimations in the No-condition, the observed anchoring effects are potentially lower. Moreover, the interaction effect might be lower and tending more towards reflecting an attenuated rather than a reversed interaction. While this does not pose a problem for the detection of the anchoring effect as a main effect, it can be problematic for the detection of the interaction effect, as interaction effects structurally require higher sample sizes to reach sufficient power than main effects and if testing attenuated rather than reversed effects (Sommet et al., 2023). Unfortunately, this issue was impossible to anticipate at the time of preparing the experiment, as it was due to a bias present in participants' estimations rather than a bias in the anchors used. Moreover, the study was distributed in German language, reached a relatively young group of participants being interested enough in the specific domain of sports to take part in the study and - as distributed online - excluded persons being not able to use a computer or absolutely rejecting digital technology. As such, generalisability of our results to groups with other characteristics is limited and possible influences of e.g. cultural background on trust in AI cannot be investigated with our dataset. Finally, the online setting of the study may come with additional limitations like reduced experimental control as well as varying attention and motivation of the participants. Like for all experimental results, understanding whether they can be fully transferred to real-world situations will require field studies, which are currently lacking in the domain of algorithm aversion (Mahmud et al., 2022) but can be pursued in the future, particularly in the domain of sports (Wunderlich & Reis, 2026).

In addition to gender differences, several further questions were not specifically targeted in the current study design but would be worth considering in future research. Poor performance or obvious errors by AI or experts naturally can influence the willingness of persons to follow their estimations. Previous research suggests that the reaction to such errors is not similar for algorithmic and human advice (Dietvorst et al., 2015), thus, it will be interesting to account for or even deliberately manipulate timely development of trust in experimental designs within the sports context. Likewise, it will be relevant to understand how participants’ behaviour changes if provided with an objective assessment on the ability (i.e. accuracy) of AI and experts, when estimations are associated with personal consequences for participants (e.g. financial profit or loss when estimating correctly), when the expert is presented in a different form (i.e. participants hearing the voice and seeing the facial expressions of the expert instead of a textual information) or when considering participants from different cultural and social backgrounds. More research is also required to better understand the influence of objectively assessed expertise, age or further personal characteristics on the tendency to trust an AI or an expert within this domain.

## Supplementary Information

Below is the link to the electronic supplementary material.

Supplementary Material 1.

## Data Availability

All data and code used for the analyses are available online in the following repository: https://doi.org/10.5281/zenodo.23569510.

## Footnotes

1 Using the notation common in R package lme4 (see Bates, Mächler, Bolker, & Walker, 2015; Brown, 2021), where we implemented the model, the formula translates to this representation (and analogously for the other models): $$\begin{aligned}Percentage&=1+ Level + Source + Level: Source\\&+(1\mid\:Participant)+(1\mid\:Match)\end{aligned}$$

## Declarations

### Use of AI-based tools

An AI-based online translation tool has been used within the process of writing. Neither ChatGPT nor any other generative AI-based tool have been used in generating or reformulating any full parts of the manuscript.
