# Supplementary material for: Trusting the AI rather than the expert? Evidence from sports-related assessments in relation to the Olympic Games 2024
Source: Psychol Res. 2026 Oct 30;90(6):195. doi: 10.1007/s00426-026-02409-z (PMC13722767; doi:10.1007/s00426-026-02409-z)
Supplement: Supplementary file 1 [file 426_2026_2409_MOESM1_ESM.docx]

# Part A) Details on the probability information labelled as AI or Expert

Our strategy to derive realistic probabilities for each match as a basis for the lower and higher probability information was based on betting odds, which have been proven to be accurate predictors in sports events. Betting odds for each match, however, were not yet available at the time of designing the experiments. Thus, we obtained betting odds for the tournament winner (which were already available) as an objective assessment of team strengths. Denoting the betting odds of team 1 to n in decimal format as $o_{1}$, …, $o_{n}$, a probability estimation for team i to win the tournament (i.e. the Olympic Games) was obtained by taking the inverse of the odds and correcting for the bookmaker margin contained in the odds, as follows:

$$p_{i}=\frac{1}{o_{i}}\cdot\frac{1}{(o_{1}+\ldots+o_{n)}}$$

Having a probability estimation for each team, we derived a realistic probability estimation $p_{realistic}$ for each match by making sure that the team with a higher tournament probability also had a higher probability to win the match, that the probability difference between both teams increased with an increasing difference in the tournament winning odds and taking into account the different frequency of drawn matches in the three respective sports games (football, handball and field hockey).

Then, we transformed these realistic probabilities into slightly higher and slightly lower probabilities communicated as information in our experimental conditions. Based on the realistic match probability $p_{realistic}$, the lower and higher probability were determined by the following formula (see illustrated in Figure 1 in the manuscript):

$$p_{lower}=0.8{\cdot p}_{realistic}\cdot{1.1}^{p_{realistic}}$$

$$p_{higher}=1-0.8{\cdot(1-p}_{realistic})\cdot{1.1}^{(1-p_{realistic})}$$

The match-level betting odds were not fully available before the tournament start, but became available during the tournaments and were thus available for collection after the experiments. We use them for obtaining market-based probabilities and for testing the accuracy of our probabilities. Here, if $o_{1}$, $o_{d},o_{2}$ are the betting odds in decimal format for team 1, a draw and team 2, respectively, then in full analogy to the tournament odds, a market-based probability for team 1 (i.e. the team of interest) can be calculated as follows:

$$p_{market}=\frac{1}{o_{1}}\cdot\frac{1}{(o_{1}+o_{d}+o_{2)}}$$

Finally, from the experiment, we could obtain the average probability estimation of all participants in the benchmark condition (no-condition), which we denote as $p_{benchmark}$. Having these three sets of probabilities as well as the actual outcome of each match, we assessed whether the accuracy in our own probability estimation had actually been accurate enough for our purposes. This was done by calculating several numbers summarised in Table A. In particular, we calculated the average value of $p_{realistic}$, $p_{market}$ and $p_{benchmark}$ to detect potential biases; the root mean squared error (RMSE, lower means better) between the different sets of probability estimations as well as the RMSE between the three sets of probability estimations and the actual match outcomes. We found no bias in the order of magnitude of the probability estimations in relation to the market (average own estimation: 44.65%; average market estimation: 44.83%) and reasonable similarity between own and market estimation with a RMSE of 12.08, Moreover, the RMSE of the own estimation to the actual match outcomes (50.97) was comparable to the one for the market estimations (51.40). All measures indicate that the own estimation was more accurate than the estimations of the participants in the No-Condition (average estimation: 52.23%, RMSE to market estimates: 17.91, RMSE to match outcomes: 55.33), which suggests that our estimations had a sufficiently high accuracy to be used in the experiments.

All betting odds information was collected from freely available online resources such as <https://www.oddsportal.com/> or <https://oddspedia.com/>. Analyses are based on 67 matches as betting odds were not available (even retrospectively) for 3 field hockey matches considered in the field hockey experiment.

Table A: Accuracy tests for the realistic probabilities used in the experiments

| Average probability estimation (in %) | | RMSE between types of probabilities (in %) | | RMSE between probabilities (in %) and outcome (accuracy) | |
| --- | --- | --- | --- | --- | --- |
| Market | 44.83 | Market vs. Benchmark | 16.88 | Market | 51.40 |
| Realistic | 44.65 | Market vs. Realistic | 12.08 | Realistic | 50.97 |
| Benchmark | 52.23 | Benchmark vs. Realistic | 17.91 | Benchmark | 55.33 |

# Part B) Details on the simulation-based power analysis

In the specific design and for the specific mixed models chosen, a conventional power analysis was not possible. First, the design required distribution of the study link quite close to the first match and its end was predefined by the tournament start. Thus, in contrast to most experimental studies, we did not have full influence on the number of participants, i.e. were unable to leave the experiment open until we reach a desired sample size. Second, analysis of power in linear mixed models not only requires assumptions on effects, but also on random and residual variances and needs to consider the exact nested structure of the data. Thus, we make use of a simulation-based power analysis, estimating the number of correctly rejected null hypotheses in simulations based on predefined properties like data structure, sample sizes, effects and variances. For our models, we require assumptions on the number of participants, number of matches (i.e. questions) per participant, Intercept, effects of Level, Source and Interaction, as well as standard deviations for the residuals and random effects of Participant and Match (see Table B). We assumed a realistic number of participants (100 for each sport, 300 in total, which is close to the actual number of participants included in the end) and analysed the power for various possible scenarios. In these scenarios, we deliberately vary those factors theoretically having the highest influence on the power, namely the relevant effect and the residual standard deviation.

Table B: Assumptions used for the simulation-based power analysis.

| **Assumption** | **Percentage (Hyp 1 and 2)** | **Confidence (Hyp 3)** |
| --- | --- | --- |
| Number of participants | 300 | 300 |
| Number of matches per participant | 24 (x 4/5 to account for the no-condition) | 24 (x 4/5 to account for the no-condition) |
| Intercept | 45% | 3.5 |
| Effect Level | 3% (testing range from 1% to 3%) | 0 |
| Effect Source | 0% | 0.2 (testing range from 0.05 to 0.25) |
| Effect Interaction | 2% (testing range from 1% to 3%) | - |
| Residual SD | 10% (testing range from 5% to 15%) | 1.0 (testing range from 0.5 to 1.5) |
| Random SD (Participants) | 5% | 1.0 |
| Random SD (Matches) | 25% | 1.0 |

Figure A: Results of the simulation-based power analysis. The x axis refers to the effect of interest, the y axis refers to the estimated power (i.e. probability to correctly reject the null hypothesis) and different residual standard deviations are illustrated by different symbols. Error bars refer to 95% confidence intervals.

Figure A shows results of the simulation-based power analysis using 250 simulations per scenario. It indicates that in most scenarios power exceeds the common benchmark of 80%. With regard to the effect of Level on Percentage (Hypothesis 1), our statistical design would be able to detect effects being as small as one tenth of the residual SD with sufficient power. The same is true for the effect of Source on Confidence (Hypothesis 3). For the interaction effect on Percentage (Hypothesis 2), at least effects as small as one fifth of the residual SD would be detected with sufficient power. Based on these analyses, we considered the design to have sufficient power to detect relevant effects.

# Part C) Details on interindividual differences

Table 2 in the manuscript reports results for chi squared tests investigating the relationship of several variables (more specifically: between personal characteristics and participants’ answers). Here, we additionally report tables of frequencies for each combination of variables in order to make the tendencies clearly visible (e.g. whether men or women tended towards AI or expert in the most helpful information). Please note that in Table C, we dichotomize expertise and age for reasons of simplicity. In comparison to the results in the manuscript, test conclusions and p values remain virtually unchanged (gender vs. self-assessed expertise: p < .001; gender vs. helpful information: p <.05; age vs. helpful information: p <.01; age vs. expertise: p = .073). Thus, this additional test can serve as a robustness test for the results shown in the manuscript.

Table C: Results of statistically analyzing interindividual differences. Results refer to $\chi^{2}$-tests testing relationships between answers of participants and personal characteristics.

|  |  | Most helpful information | | | Self-assessed expertise | |
| --- | --- | --- | --- | --- | --- | --- |
|  |  | AI | Expert | None | Higher (>= 7) | Lower (<=6) |
| Gender | Male | 42 (21.9%) | 51 (26.6%) | 99 (51.6%) | 119 (61.0%) | 76 (39.0%) |
|  | Female | 5 (7.0%) | 27 (38.0%) | 39 (54.9%) | 29 (38.7%) | 46 (61.3%) |
| Age | Older (>= 30) | 10 (9.3%) | 32 (29.9%) | 65 (60.7%) | 54 (48.6%) | 57 (51.4%) |
|  | Younger (<30) | 37 (23.4%) | 47 (29.7%) | 74 (46.8%) | 96 (59.6%) | 65 (40.4%) |

# Part D) Detailed results of Explorative Analyses

Table 5 in the manuscript summarises results of the exploratory analyses, yet only restricted to the model estimate for the effect relevant to the respective hypotheses and the respective p-value. Table D summarises the full results of analysis, where the upper part summarises all model estimates when fitting the full model for Probability as well as the full results of likelihood ratio tests when comparing the model fits of the full model to the restricted model (Hyp. 2) and of the restricted model to the null model (Hyp. 1). The lower part summarises all model estimates when fitting the full model for Confidence as well as the full results of likelihood ratio tests when comparing the full model to the null model (Hyp. 3).

Table D: Full results of explorative analyses repeating the statistical analyses described in the manuscript for specific subgroups of the participants. The upper part of the Figure refers to analysis of Probability (Hyp. 1 and 2), while the lower part refers to analyses of Confidence (Hyp. 3). Please note that N to the number of individual estimations of a match.

| **Probability (Hypothesis 1 and Hypothesis 2)** | | | | | | | | |
| --- | --- | --- | --- | --- | --- | --- | --- | --- |
| Subgroup | | N | Intercept | Level | Source | Interaction | LR-Test Level | LR-Test Interaction |
| All | | 4948 | 49.92 | 4.75 | 0.33 | -1.32 | $\chi^{2}(1)$=103.12, p < .001* | $\chi^{2}(1)$=2.01, p = .156 |
| Sport | Football | 1253 | 50.52 | 4.23 | -0.003 | -3.12 | $\chi^{2}(1)$=17.04, p < .001* | $\chi^{2}(1)$=2.32, p = .128 |
|  | Handball | 1814 | 50.69 | 2.95 | 0.18 | 0.43 | $\chi^{2}(1)$=14.20, p < .001* | $\chi^{2}(1)$=0.08, p = .781 |
|  | Field Hockey | 1881 | 48.50 | 6.69 | 0.67 | -1.70 | $\chi^{2}(1)$=94.62, p < .001* | $\chi^{2}(1)$=1.57, p = .210 |
| Gender | Female | 1322 | 49.94 | 4.50 | 1.77 | -0.20 | $\chi^{2}(1)$=20.62, p < .001* | $\chi^{2}(1)$=0.01, p = .921 |
|  | Male | 3590 | 49.92 | 4.79 | -0.11 | -1.49 | $\chi^{2}(1)$=81.45, p < .001* | $\chi^{2}(1)$=1.99, p = .158 |
| Expertise | Higher (>= 7) | 2755 | 49.59 | 4.13 | 0.17 | -1.68 | $\chi^{2}(1)$=45.61, p < .001* | $\chi^{2}(1)$=1.88, p = .170 |
|  | Lower (<= 6) | 2193 | 50.15 | 5.86 | 0.93 | -0.47 | $\chi^{2}(1)$=66.76, p < .001* | $\chi^{2}(1)$=0.11, p = .740 |
| Helpful Information | AI | 858 | 48.84 | 8.64 | 1.02 | -5.36 | $\chi^{2}(1)$=81.16, p < .001* | $\chi^{2}(1)$=7.97, p < .01* |
|  | Expert | 1426 | 49.51 | 5.88 | 0.85 | -0.23 | $\chi^{2}(1)$=41.58, p < .001* | $\chi^{2}(1)$=0.02, p = .901 |
|  | None | 2589 | 50.06 | 2.70 | -0.26 | -0.64 | $\chi^{2}(1)$=16.53, p < .001* | $\chi^{2}(1)$=0.24, p = .627 |
| Age Group | < 30 | 2970 | 49.26 | 5.29 | 0.77 | -0.80 | $\chi^{2}(1)$=85.10, p < .001* | $\chi^{2}(1)$=0.49, p = .482 |
|  | >= 30 | 1978 | 50.94 | 3.62 | -0.27 | -1.77 | $\chi^{2}(1)$=20.83, p < .001* | $\chi^{2}(1)$=1.24, p = .265 |
| **Confidence (Hypothesis 3)** | | | | | | | | |
| Subgroup | | N | Intercept | Level | Source |  | LR-Test Source | |
| All | | 5006 | 4.20 | 0.02 | -0.10 |  | $\chi^{2}(1)$ = 18.65, p < .001* | |
| Sport | Football | 1267 | 4.00 | 0.02 | -0.02 |  | $\chi^{2}(1)$ = 0.19, p = .663 | |
|  | Handball | 1853 | 4.40 | 0.04 | 0.04 |  | $\chi^{2}(1)$ = 1.14, p = .286 | |
|  | Field Hockey | 1886 | 4.17 | 0.001 | -0.29 |  | $\chi^{2}(1)$ = 60.86, p < .001* | |
| Gender | Female | 1347 | 3.95 | 0.03 | 0.01 |  | $\chi^{2}(1)$ = 0.06, p = .814 | |
|  | Male | 3621 | 4.30 | 0.01 | -0.14 |  | $\chi^{2}(1)$ = 27.79, p < .001* | |
| Expertise | Higher (>= 7) | 2779 | 4.51 | 0.01 | -0.11 |  | $\chi^{2}(1)$ = 13.92, p < .001* | |
|  | Lower (<= 6) | 2227 | 3.85 | 0.05 | -0.08 |  | $\chi^{2}(1)$ = 4.76, p < .05* | |
| Helpful Information | AI | 863 | 4.09 | -0.02 | -0.30 |  | $\chi^{2}(1)$ = 24.93, p < .001* | |
|  | Expert | 1441 | 4.01 | 0.05 | -0.08 |  | $\chi^{2}(1)$ = 3.22, p = .073 | |
|  | None | 2625 | 4.37 | 0.03 | -0.04 |  | $\chi^{2}(1)$ = 1.70, p = .193 | |
| Age Group | < 30 | 2986 | 4.30 | 0.05 | -0.15 |  | $\chi^{2}(1)$ = 27.52, p < .001* | |
|  | >= 30 | 2020 | 4.10 | -0.01 | -0.02 |  | $\chi^{2}(1)$ = 0.20, p = .652 | |

* statistically significant effects
